# Photon-counting CT allows better visualization of temporal bone structures in comparison with current generation multi-detector CT

**DOI:** 10.1186/s13244-023-01467-w

**Published:** 2023-07-03

**Authors:** Robert Hermans, Lukas Boomgaert, Lesley Cockmartin, Joke Binst, Rashèl De Stefanis, Hilde Bosmans

**Affiliations:** 1 Department of Radiology, University Hospitals Leuven, Herestraat 49, 3000 Leuven, Belgium; 2 Department of Imaging and Pathology, KU Leuven-University of Leuven, Leuven, Belgium

**Keywords:** Computed tomography, Temporal bone imaging, Photon-counting CT, Radiation dose

## Abstract

**Purpose:**

To compare photon-counting CT (PCCT) and multi-detector CT (MDCT) for visualization of temporal bone anatomic structures.

**Methods:**

Thirty-six exams of temporal bones without pathology were collected from consecutive patients on a MDCT, and another 35 exams on a PCCT scanner. Two radiologists independently scored visibility of 14 structures for the MDCT and PCCT dataset, using a 5-point Likert scale, with a 2-month wash-out period. For MDCT, the acquisition parameters were: 110 kV, 64 × 0.6 mm (slice thickness reconstructed to 0.4 mm), pitch 0.85, quality ref. mAs 150, and 1 s rotation time; for PCCT: 120 kV, 144 × 0.2 mm, pitch 0.35, IQ level 75, and 0.5 s rotation time. Patient doses were reported as dose length product values (DLP). Statistical analysis was done using the Mann–Whitney U test, visual grading characteristic (VGC) analysis, and ordinal regression.

**Results:**

Substantial agreement was found between readers (intraclass correlation coefficient 0.63 and 0.52 for MDCT and PCCT, resp.). All structures were scored higher for PCCT (*p* < 0.0001), except for Arnold’s canal (*p* = 0.12). The area under the VGC curve was 0.76 (95% CI, 0.73–0.79), indicating a significantly better visualization on PCCT. Ordinal regression showed the odds for better visualization are 354 times higher (95% CI, 75–1673) in PCCT (*p* < 0.0001). Average (range) of DLP was 95 (79–127) mGy*cm for MDCT and 74 (50–95) mGy*cm for PCCT (*p* < 0.001).

**Conclusion:**

PCCT provides a better depiction of temporal bone anatomy than MDCT, at a lower radiation dose.

**Graphical Abstract:**

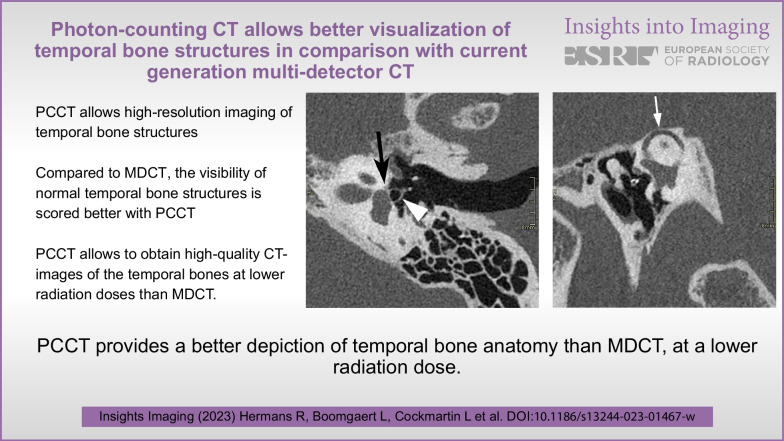

**Critical relevance statement:**

PCCT provides a better depiction of temporal bone anatomy than MDCT, at a lower radiation dose.

**Key points:**

1. PCCT allows high-resolution imaging of temporal bone structures.
2. Compared to MDCT, the visibility of normal temporal bone structures is scored better with PCCT.
3. PCCT allows to obtain high-quality CT images of the temporal bones at lower radiation doses than MDCT.

## Introduction

Multi-detector CT (MDCT) scanners use energy-integrating detectors, providing signal proportional to the total photon energy. The size of the detector elements is a major factor that limits the spatial resolution of such systems [1]. In a photon-counting CT (PCCT) scanner, the detector counts individual photons while measuring their energy. Such detectors have several advantages, such as less impact of electronic noise, multi-energy imaging, a better contrast to noise ratio, and also a better geometric efficiency as there are no septa between detector elements (such as in MDCT) [1, 2]. Imaging of the temporal bone, containing many delicate anatomical structures, such as the auditory ossicles among others, could benefit from the increased spatial resolution provided by PCCT. To date the advantage of PCCT for temporal bone imaging has been demonstrated in a cadaver study [1], as well as in preliminary clinical visual grading studies on small numbers of patients [3, 4]. The study of Zhou et al. [1] compared MDCT and PCCT high-resolution images of ten cadaveric temporal bone specimens using objective noise measurements (i.e., standard deviation of the Hounsfield Units in soft tissue) and subjective image preference ranking. The PCCT images showed significantly lower noise than MDCT together with a ranking preference for the PCCT images at equivalent dose levels. Differences were most obvious for the visibility of the modiolus. Benson et al. [4] conducted a side-by-side visual grading study on patients undergoing both a PCCT and MDCT exam of the temporal bone. They found a significant improvement in image quality and visualization of critical structures with a dose reduction of 31% for PCCT. Scores for oval window and incudostapedial joint were the highest for PCCT.

The purpose of this study was to compare MDCT with PCCT exams for the visualization of normal temporal bone anatomical structures in a series of patients in standard clinical practice, and to compare the radiation dose needed for acquiring these exams.

## Material and methods

This prospective, single-center study was approved by the Ethics Committee of the University Hospitals Leuven (reference S65765). Historical scans of consecutive temporal bone exams performed on MDCT during 3 months (September–November 2021) were collected. Patients undergoing a CT exam of the temporal bone on the PCCT scanner were prospectively recruited during 3 months (April–June 2022), and informed consent was obtained.

For the MDCT scanner (Siemens Somatom Force, Siemens Healthineers, Forchheim, Germany), the acquisition parameters were as routinely used in clinical practice: 110 kV, 64 × 0.6 mm, pitch 0.85, quality ref. mAs 150, and 1 s rotation time; the images were reconstructed with a thickness of 0.4 mm by combining a smaller focal spot, comb filter and a z-axis deconvolution technique [5]. For the PCCT scanner (Siemens Naeotom Alpha, Siemens Healthineers, Forchheim, Germany), the acquisition parameters were 120 kV, 144 × 0.2 mm, pitch 0.35, IQ level 75, and 0.5 s rotation time. These parameters were determined based on an earlier pilot study on a cadaver head, determining the lowest radiation dose yielding an acceptable image quality. The image data were reconstructed using a high-resolution kernel.

Thirty-six exams of temporal bones without pathology, as judged by a senior head and neck radiologist (31 years of experience), were collected (*n* = 26; 12 female, 14 male patients; average age = 41 years (range 14–76)) on MDCT. Similarly, thirty-five exams of temporal bones without pathology were collected on the PCCT scanner (*n* = 31; 18 female, 13 male patients; average age = 51 years (range 16–79)).

A month later, the senior and a second staff head and neck radiologist (4 years of experience) independently scored visibility of 14 normal anatomical structures (Table 1) for the MDCT and PCCT dataset separately using the open-source software ViewDEX 3.0 [6–8]. Apart from the native images, multiplanar reformation (MPR) images in the axial plane (parallel to the lateral semicircular canal) and coronal plane (perpendicular to the axial reformattings) with a slice thickness of 0.4 mm were also evaluated. A 5-point Likert scale was used to score the anatomical structures as invisible (1), difficult to recognize (2), recognizable (3), clearly recognizable (4) or as excellent visibility (5). The scoring of these structures on MDCT and PCCT studies was done with a 2-month wash-out period. Inter-reader agreement was assessed using intraclass correlation coefficient (ICC; two-way random effects, single measure). Further analysis was done on the median score of both readers. Visual grading scores were compared between MDCT and PCCT using a Mann–Whitney U test for each structure separately (Bonferroni correction: *α* = 0.0033). VGC analysis was used to compare overall anatomy visualization between both scanners; ordinal regression quantified the odds of better overall visibility in PCCT versus MDCT. Patient doses were reported as DLP values and compared using a Mann–Whitney U test. Statistical tests were performed using SPSS version 28 (IBM, Armonk, USA).

**Table 1:** Anatomic structures investigated

Tympanic membrane	
Lateral malleal ligament	
Incudostapedial joint	
Stapedial crura	
Head of stapes	
Stapedius muscle tendon	
Cochlear modiolus	
Cochlear lamina spiralis	
Chorda tympani canal	
Proximal intratympanic course of chorda tympani	
Distal intratympanic course of chorda tympani	
Jacobson’s canal	
Arnold’s canal	
Cortical lining of tympanic segment facial nerve canal	

## Results

**Fig. 1:**
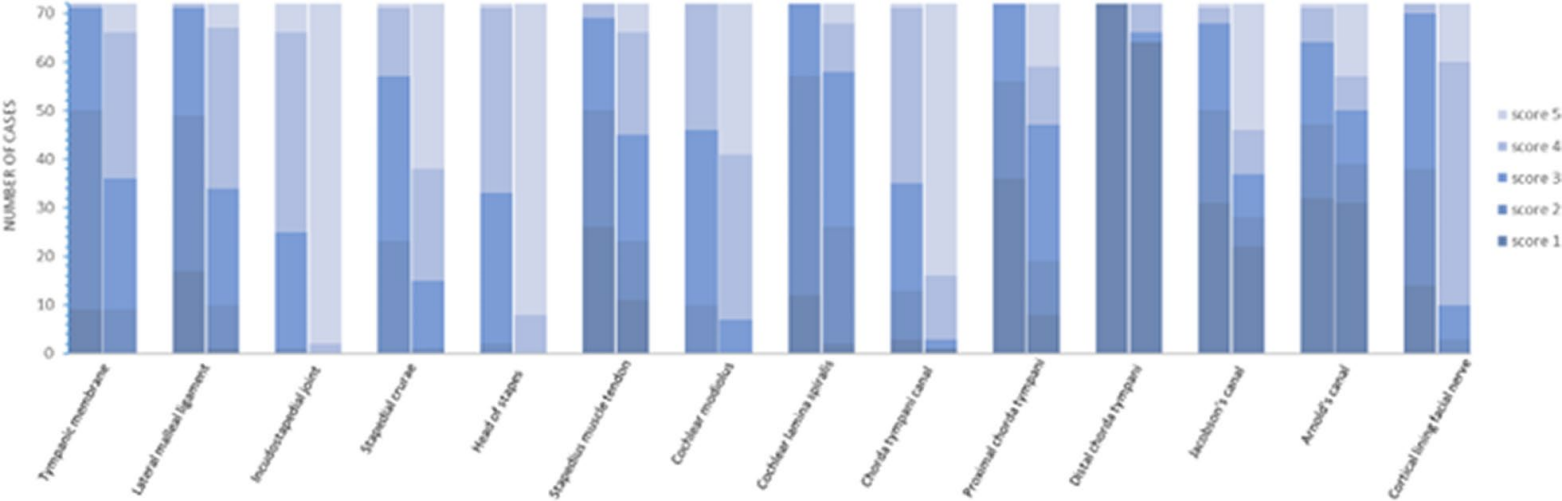
Frequency histogram showing the individual scores for both readers (left column = MDCT, right column = PCCT)

Figure 1 shows the frequency histogram for the individual visual grading scores for each reader. Substantial agreement was found between readers (ICC = 0.63 (95% CI, 0.57–0.68) and 0.52 (95% CI, 0.30–0.66) for MDCT and PCCT. resp.).

**Fig. 2:**
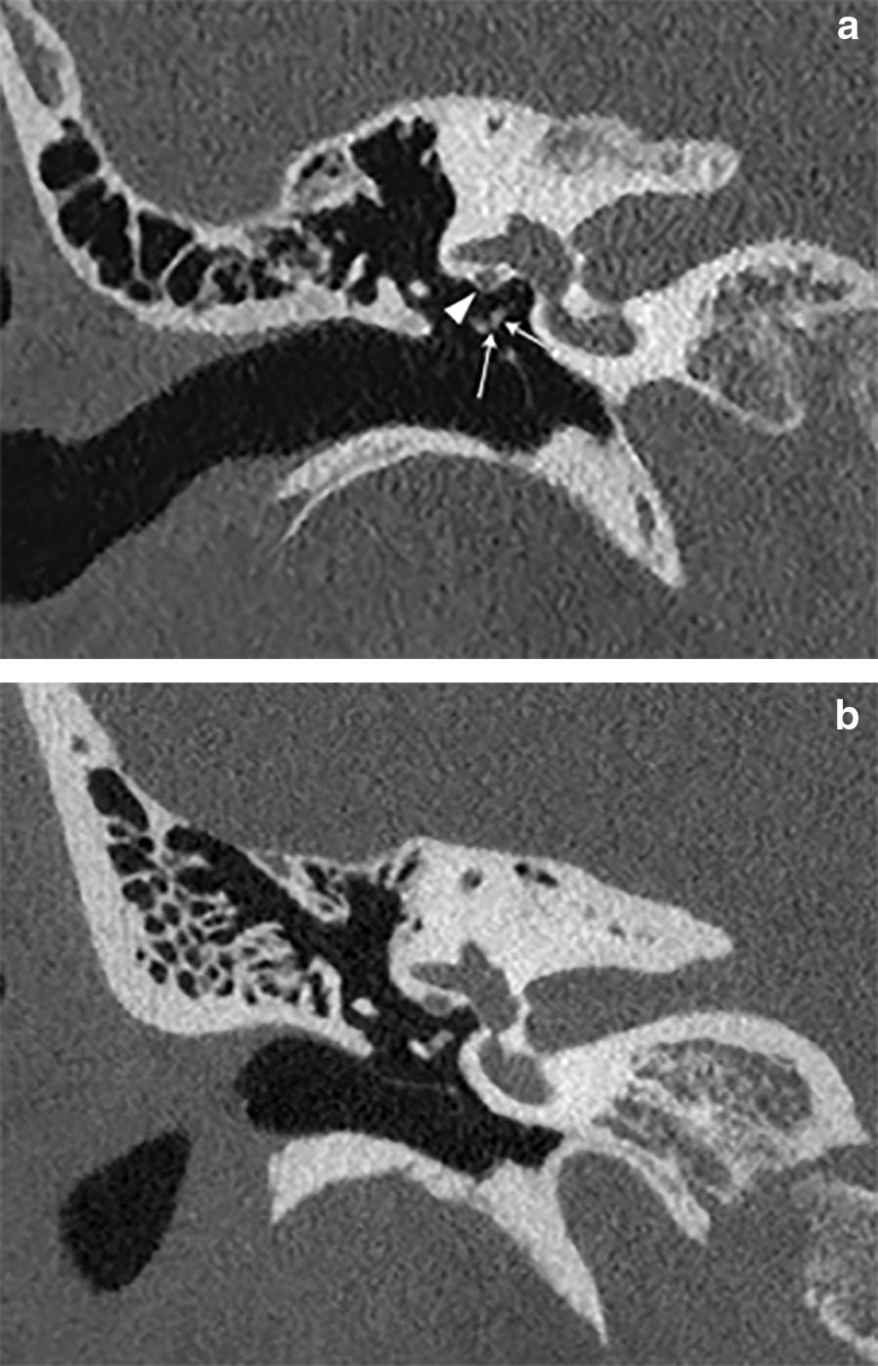
Coronal reformatted MDCT (**a**) and PCCT (**b**) image through right temporal bone. Incudostapedial joint (left arrow), stapedial head (right arrow) and cortical lining of tympanic segment of facial nerve canal (arrowhead) are labeled. In these patients, these structures were on MDCT scored by both readers as ‘recognizable’ (score 3) or ‘clearly recognizable’ (score 4), while on PCCT, both readers scored these as ‘excellent visibility’ (score 5)

**Fig. 3:**
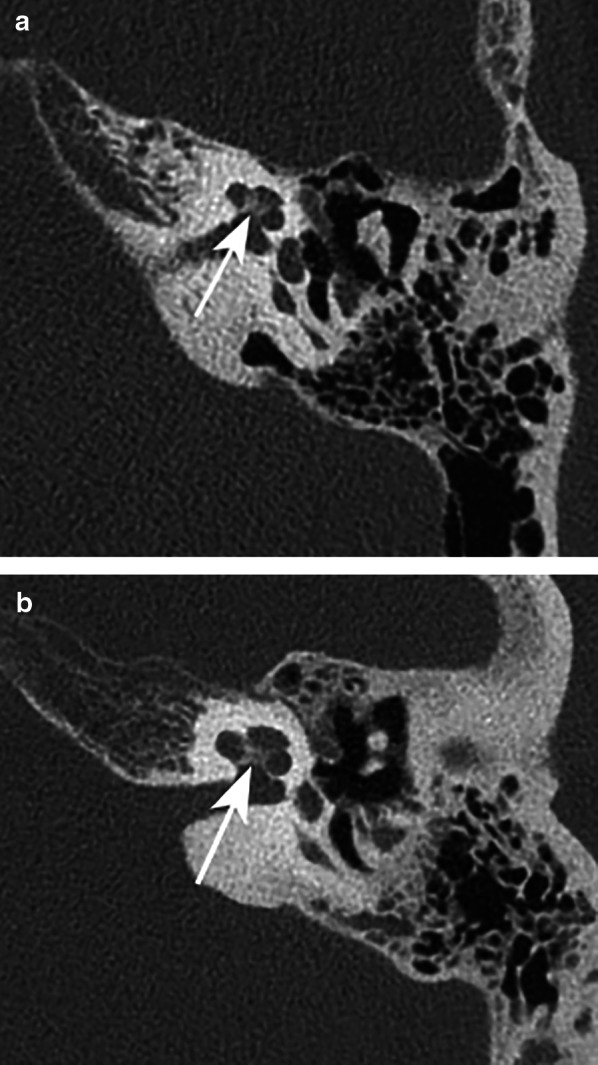
Axial reformatted MDCT (**a**) and PCCT (**b**) image through left temporal bone. In both images, the cochlear modiolus (arrow) is visible, and scored as ‘recognizable’ (score 3) or ‘clearly recognizable’ (score 4) on MDCT. On PCCT, the modiolus was scored as ‘clearly recognizable’ (score 4) and ‘excellent visibility’ (score 5), respectively

In 13 out of 14 evaluated structures, the scoring was higher for PCCT (*p* < 0.0001). The difference in scoring was more pronounced for structures such as the tympanic membrane, stapedial head, incudostapedial joint, cortical margin of the tympanic segment of the facial nerve (Fig. 2), and cochlear modiolus (Fig. 3). In others, such as the chorda tympani canal, intratympanic course of the chorda tympani and Jacobson’s canal, the difference in scoring was less pronounced yet still significant (*p* < 0.0001). Only for Arnold’s canal, no significant difference was found (*p* = 0.12). Overall, structures were scored higher on PCCT in 68%, equal in 19%, and less in 5% of judgments.

**Fig. 4:**
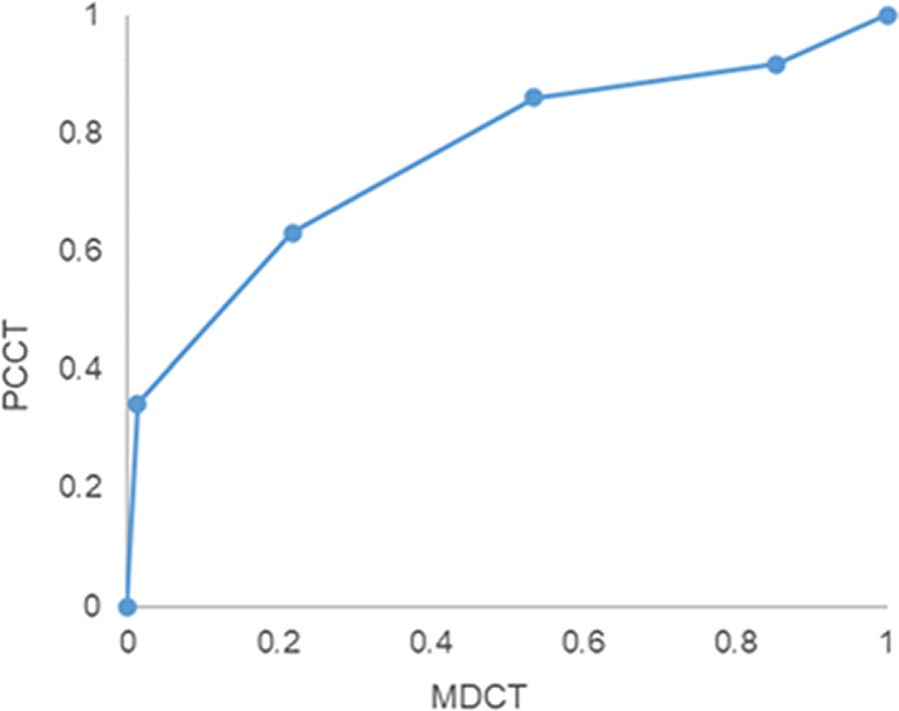
Visual grading characteristic (VGC) curve comparing scoring of temporal bone structures on MDCT and PCCT (AUC = 0.76; 95% CI [0.73–0.79])

The area under the VGC curve (AUC) was 0.76 (95% confidence interval (CI), 0.73–0.79), indicating a significant better visualization on PCCT (Fig. 4). Ordinal regression showed the odds for better visualization are 354 times higher (95% CI, 75–1673) in PCCT compared to MDCT (*p* < 0.0001).

**Fig. 5:**
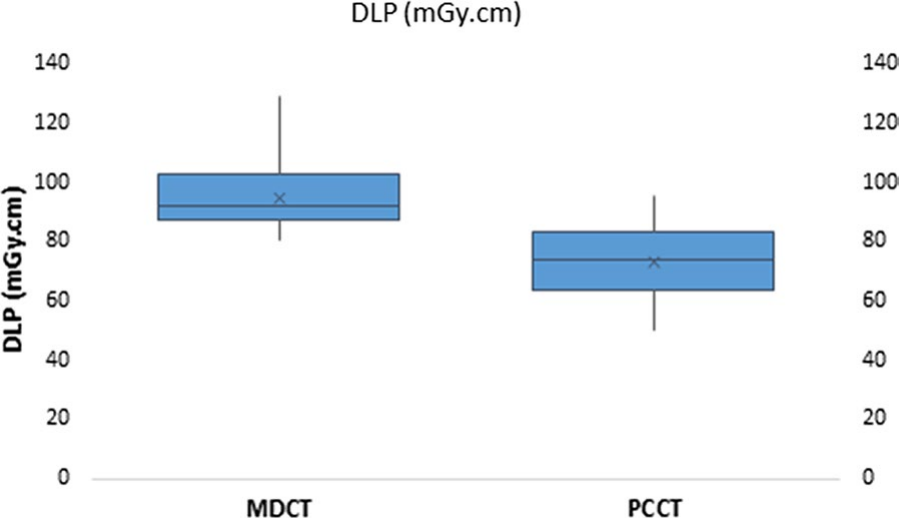
Distribution of DLP on MDCT and PCCT exams (*p* < 0.001)

Average (range) of DLP was 95 (79–127) mGy*cm for MDCT and 74 (50–95) mGy*cm for PCCT (*p* < 0.001) (Fig. 5).

## Discussion

The study reports on the image quality of PCCT exams of the temporal bone in clinical routine and compares it with historical standard MDCT exams. The results are in line with what can be expected based on the physical characteristics of a PCCT machine: the high spatial resolution with 0.2-mm slice thickness offered by such a system allows better visualization of small anatomical structures within the temporal bones compared to the current high-end MDCT with 0.4-mm slice thickness.

Only for Arnold’s canal, no significant difference was found between MDCT and PCCT. This is likely due to the variable and inconsistent appearance of Arnold's and Jacobson's canals on CT exams [9], partly related to the variable bone pneumatization surrounding these canals, as well as to complex variations of the nerves themselves [10]. Also using PCCT, identification of these canals is sometimes challenging, and these canals cannot always be confidently identified.

This study evaluated normal anatomical structures in the temporal bones. Whether PCCT also allows to better evaluate pathological changes in the temporal bones was not investigated. However, based on the findings in this study, it can be anticipated that PCCT, based on its higher spatial resolution, will allow to identify more confidently subtle abnormalities, such as for example very discrete forms of fenestral otosclerosis [11] or third window pathology [12].

**Fig. 6:**
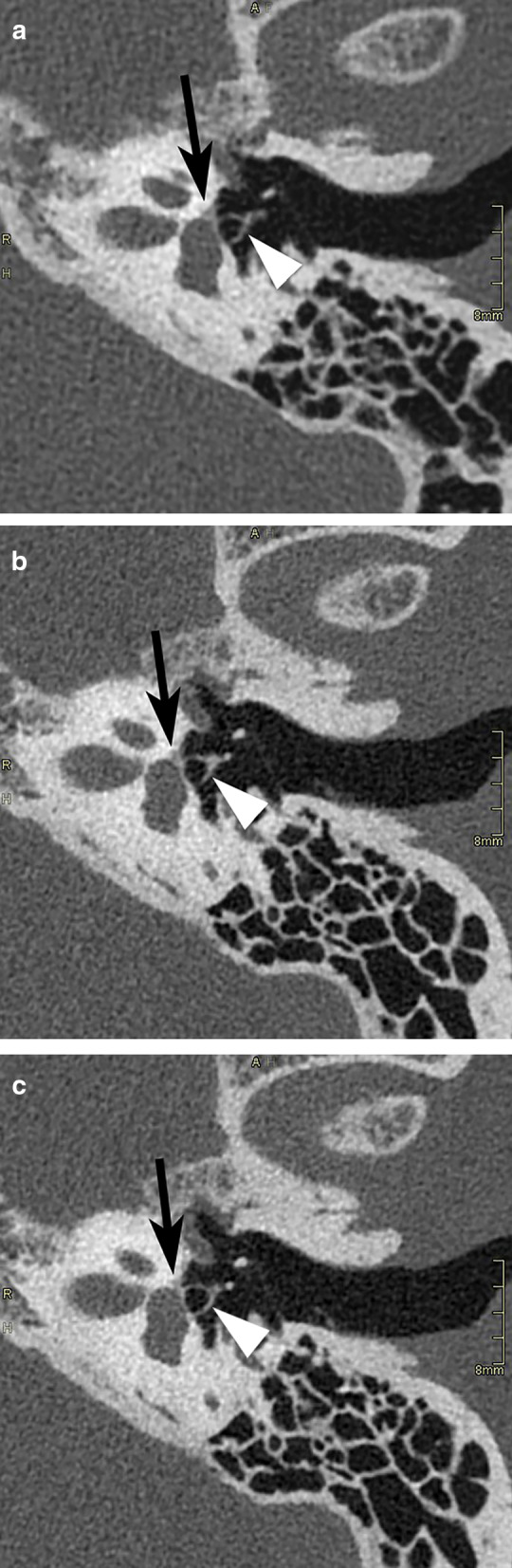
MDCT (**a**) and PCCT (**b**, **c**) images reformatted in the axial stapes plane, obtained in the same patient, 6 years apart, using the same acquisition parameters as in this study. Subtle lucency just anterior to the footplate, corresponding to fenestral otosclerosis (arrows), on the PCCT study because of the thinner slice thickness visible on two adjacent slices, potentially increasing reader confidence. On the PCCT images, the stapedial superstructure (arrowheads) is slightly better visible

**Fig. 7:**
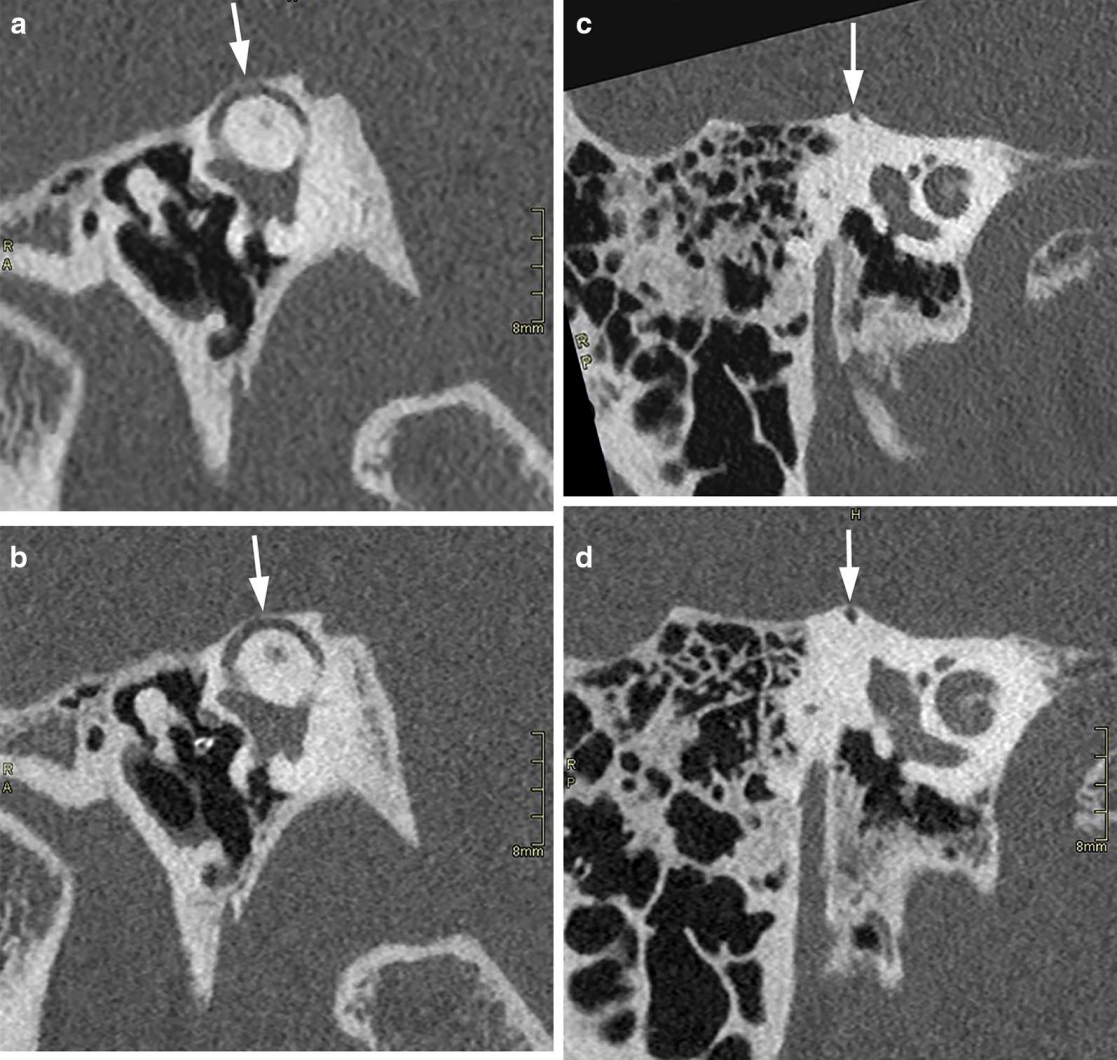
Same patient as in Fig. 6. MDCT (**a**–**c**) and PCCT (**b**–**d**) images reformatted according to Pöschl (**a**, **b**) and Stenvers (**c**, **d**) plane. The bony layer covering the superior semicircular canal (arrows) is better visible on the PCCT images

**Fig. 8:**
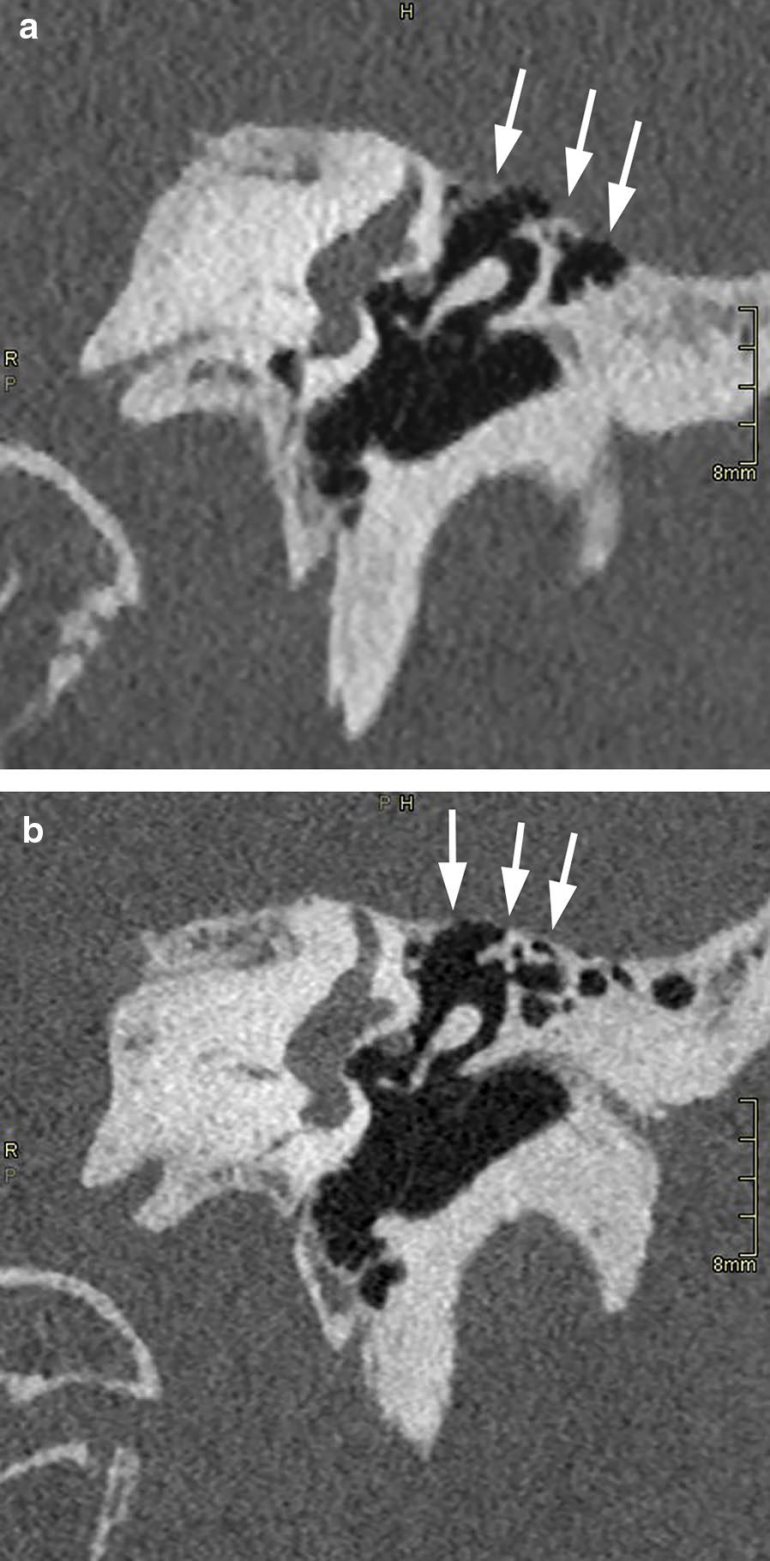
Same patient as in Figs. 6 and 7. MDCT (**a**) and PCCT (**b**) image reformatted in the coronal plane. On MDCT, the very thin tegmen tympani (arrows) is not clearly visible; on PCCT, the tegmen is visible as an intact bony line

For methodological reasons, in this study the images routinely available were used to compare MDCT with PCCT. In selected cases, additional reformatting in other planes, such as in the Stenvers and Pöschl planes [13], or in the axial stapes plane [14] can be useful to better depict certain anatomical structures. As PCCT allows to acquire thinner native slices than MDCT, higher-quality reformattings are possible, likely further accentuating the advantage of this technique. This is illustrated in Figs. 6, 7 and 8, showing images in a patient (not included in the current study), in whom for clinical reasons on two separate time points, a MDCT and PCCT study was obtained, using the same acquisition parameters as in this study.

The use of visual grading analysis for performance testing has a long tradition in X-ray imaging and CT; if a modality shows normal anatomy very well, it is expected that abnormal structures will follow. Moreover, this approach uses images that are readily available without recruitment of specific pathology cases. The European Guidelines on quality criteria provide standards for visual grading criteria for different anatomy on CT exams [15]. Visual grading characteristic analysis and ordinal regression are scientifically accepted methods for assessing clinical image quality based on ordinal scores [16, 17] and have been applied previously for the optimization of CT head examinations [18]. In this study, we have applied visual grading characteristics analysis to compare MDCT and PCCT giving all image quality criteria an equal weight and found a significant better quality grading for PCCT. In addition, ordinal regression showed how much better PCCT was rated compared to MDCT (odds ratio = 354) independent of the reader. Both Zhou et al. [1] and Benson et al. [4] have applied visual grading to high-resolution images of the temporal bone in cadavers and patients, respectively. They compared ultrahigh-resolution PCCT and MDCT images and found a significant preference for the PCCT images. The study of Benson et al. [4] uses the same MDCT scanner (Siemens Somatom Force) for comparison, whereas the PCCT scanner is the prototype scanner Siemens Count Plus, whereas our study is performed on the next-generation commercial scanner Siemens Naeotom Alpha. Although final results of Benson et al. [4] were the same as in our study, the chosen critical structures were slightly different as well as the analysis methods. Furthermore, studies on other high-resolution applications on PCCT scanners are providing similar results. Wehrse et al. [19] showed an improved visualization of size and margins of bone metastases on PCCT versus MDCT. In an experimental study, Ruetters et al. [20] evaluated PCCT for dental imaging showing a preference of PCCT versus cone-beam CT (CBCT) for the visibility of structures such as root canal, spongious bone, and cortical bone. The commercialization of the PCCT scanner will likely expand the number of studies on high-resolution applications in short time.

Technical studies could have paralleled this subjective study. Rajendran et al. [3] combined phantom and technical measurements with clinical measurements on one patient for temporal bone imaging. Patient dose measurements identified a 37% decrease in radiation dose for PCCT accompanied with a 46% reduction in image noise [3]. Phantom measurements included HU accuracy, noise power spectrum, modulation transfer function (resolution) measurements and material decomposition. The technical comparison of the performance of different modalities in terms of resolution and noise power is however challenging and goes beyond the scope of this paper. Ideally task-based detectability metrics are applied for critical structures for the modality and clinical indication under investigation. This is yet to be worked out for PCCT as well as for temporal bone imaging in general.

The improved quality for a higher resolution, yet lower dose acquisition, is surprising as improved quality is usually associated with higher radiation doses. Klein et al. showed that it is the result of the small pixels below the resolution limit of the PCCT system that makes it clinically possible to achieve equal or better image quality at lower dose levels [21].

In this study, the average radiation dose was 26% less for PCCT compared to MDCT; this compares well with the average 31% lower dose reported in another study [4]. The acquisition parameters on MDCT and PCCT used in this study are based on a pilot study on a cadaver head, before both machines were clinically used. The preprogrammed dose settings on the PCCT scanner are now characterized by an image quality metrics (IQ level) rather than an (effective) mAs. A fixed IQ level is said to guarantee the same quality on all the latest Siemens scanners. Our Siemens Force CT scanner did however not run the latest software, and, as far as we know, the value of the IQ level remains to be tested. During such pilot study, the threshold radiation dose still yielding an acceptable signal-to-noise ratio and overall image quality was determined by trial and error. As evaluation of such pilot images is to some extent subjective, further radiation dose reduction without losing diagnostic information might still be possible.

For MDCT, an earlier study from our group indicated that the radiation dose in temporal bone MDCT can be as low as in CBCT [22]. As found in the current study, PCCT allows to reach these lower levels of radiation dose comparable to CBCT. Although high-end CBCT-machines are able to provide images with even thinner slice thickness, an advantage of PCCT over CBCT is that also soft tissue evaluation is possible at low radiation dose, which is of value in a number of indications. A study comparing PCCT with a recent high-end CBCT machine is currently, to our knowledge, not yet available for temporal bone imaging.

A limitation of this study is that the MDCT and PCCT exams were judged in separate sessions in a non-blinded manner. Blinding of these exams was not possible, as the technical image quality allows to recognize on which machine these were acquired. Therefore, it cannot be excluded that the results are influenced by some observer bias, possibly in favor of PCCT.

## Conclusion

PCCT provides a better depiction of temporal bone anatomy than MDCT, at a lower radiation dose. Based on the findings in this study, it can be anticipated that PCCT will allow to identify with more confidence subtle abnormalities in the temporal bones, less well seen on MDCT, and therefore contribute to a better management of patients suffering from ear pathologies.

## Data Availability

All data generated in this study are reported in this manuscript.

## Abbreviations

AUC: Area under curve
CBCT: Cone-beam CT
CI: Confidence interval
DLP: Dose length product
ICC: Intraclass correlation coefficient
MDCT: Multi-detector CT
MPR: Multiplanar reformation
PCCT: Photon-counting CT
VGC: Visual grading characteristic
